# Dynamics analysis and optimal control of SIVR epidemic model with incomplete immunity

**DOI:** 10.1186/s13662-022-03723-7

**Published:** 2022-07-18

**Authors:** Yiming Liu, Shuang Jian, Jianguo Gao

**Affiliations:** School of Mathematics and Information Science, North Minzu University, Yinchuan, 750021 P.R. China

**Keywords:** SIVR model, Well-posedness, Basic reproduction number, Lyapunov function, Spatial heterogeneity

## Abstract

In this paper, we establish an SIVR model with diffusion, spatially heterogeneous, latent infection, and incomplete immunity in the Neumann boundary condition. Firstly, the threshold dynamic behavior of the model is proved by using the operator semigroup method, the well-posedness of the solution and the basic reproduction number $\Re _{0}$ are given. When $\Re _{0}<1$, the disease-free equilibrium is globally asymptotically stable, the disease will be extinct; when $\Re _{0}>1$, the epidemic equilibrium is globally asymptotically stable, the disease will persist with probability one. Then, we introduce the patient’s treatment into the system as the control parameter, and the optimal control of the system is discussed by applying the Hamiltonian function and the adjoint equation. Finally, the theoretical results are verified by numerical simulation.

## Introduction

The SARS in 2003, the Zika virus (ZIKV) invasion in 2013, the H7N9 avian influenza pandemic, and the emergence of the Dengue virus in the world, these recurrent infectious diseases and various emerging infectious diseases have been challenging modern life and medical standards [1]. For example, COVID-19, which broke out in 2019, is still affecting the world. As of August 24, 2021, the cumulative number of COVID-19 cases and deaths has reached 212,357,898 and 4,439,843. Therefore, how to prevent and control the occurrence and spread of infectious diseases is one of the hot issues today.

From the perspective of mathematics, the study of infectious diseases usually starts according to the transmission mechanism of diseases, which is analyzed by establishing mathematical models. The earliest epidemic model was established by Kermack and Mckendrick. They established the plague susceptibility infection removal model (SIR) [2] in 1927 and the plague susceptible infected susceptible model (SIS) [3] in 1932, respectively. Since the establishment of SIR and SIS models, most of the subsequent research is based on the standard SIR model. Among the existing prevention and treatment methods for infectious diseases, vaccine injection is one of the fast and effective methods. For example, in the prevention and control of COVID-19, vaccine injection can reduce the infection rate of the Delta variant virus to a certain extent. Therefore, an increasing number of researchers take vaccine injection into account in the process of modeling infectious diseases to make the model close to the actual situation.

Among them, Chen et al. in [4] established the susceptibility vaccination–infection isolation–recovery (SVIQR) model and susceptibility–vaccination–infection isolation (SVIQS) model, respectively. The basic reproduction number of the two models was given. Furthermore, the global attractivity and the global asymptotic stability of the solutions were proved by the Lyapunov function method. And the existence of backward bifurcation was also proved. In [5], Kribs-Zaleta and Velasco-Hernández studied a simple SIV model with inoculation and demonstrated the backward bifurcation of solutions to some parameter values. At the same time, complete bifurcation analysis of the model was given under the condition that the vaccine reduces the basic reproduction number. Liu et al. in [6] studied the following SIVR system:

1.1 $$ \textstyle\begin{cases} S_{t}=\lambda -\beta _{1}SI-(\mu +\alpha )S, \\ V_{t}=\alpha S-\beta _{2}VI-(\mu +\gamma )E, \\ I_{t}=\beta _{1}SI+\beta _{2}VI-(\mu +\delta )I, \\ R_{t}=\gamma V+\delta I-\mu R. \end{cases} $$

Here, *λ* is the constant update rate of the susceptible host and *α* is the rate at which susceptible individuals are vaccinated. $\beta _{1}$ and $\beta _{2}$ are the transmission rates of infected people in contact with susceptible groups and vaccinated groups, respectively. Since vaccinated individuals may have partial immunity during vaccination, it is assumed that $\beta _{1}>\beta _{2}$. *μ* is the host mortality per compartment. *γ* and *δ* are the recurrence rates of vaccinated people and infected people, respectively. All of these parameters are assumed to be positive. In [6], the authors gave the threshold dynamics of system (1.1) by using the basic reproduction number, showing that reducing the number of infected individuals by vaccination can control the disease. In addition, many researchers have studied infectious disease models with immunization from the perspective of age structure [7] and pulse vaccination [8].

The above models are all established in a homogeneous space environment. However, in practice, the transmission of some diseases is often related to spatial location. For example, the transmission rate of COVID-19 in Asia is different from that in North America. In [9], Wu et al. discussed a class of spatially heterogeneous host-pathogen models. The authors used the basic reproduction number to discuss the threshold dynamic behavior of the models and gave the asymptotic behavior of the models. In [10], a reaction-diffusion model of SVIR infection in a spatially heterogeneous environment was proposed. The authors gave the proof of the extinction and persistence of the disease by giving a basic reproduction number. In [11], the authors established an SIVS epidemic model with a degree-dependent transmission rate and incomplete vaccination on a scale-free network. The global asymptotic stability of the equilibrium and the global attractivity of the unique endemic equilibrium were proved. In addition, the effects of various immunization programs such as unified immunization, target immunization, and acquaintance immunization were studied and compared.

Motivated by the recent development of epidemic modeling, the optimal control problem is often discussed in some cases, optimal control theory is one of the important branches of mathematical optimization, which is often used to study how to find a control for a dynamic system in a period of time to optimize the objective function. Thus we consider two different models based on (1.1). The first is a direct extension of (1.1). A reaction-diffusion SIVR model is established based on spatial heterogeneity with incomplete immunity. The well-posedness of the system is discussed by using the operator semigroup method. At the same time, the global dynamic behavior of the system solution is discussed by analyzing the basic reproduction number. In the second model, as [12, 13], it is assumed that the spread of a disease can be influenced by decision-makers. That is, decision-makers can control the response rate to a certain extent by increasing the treatment ability or the efficiency of drug treatment. Therefore, by further expanding the model, we obtain the control system under the assumption of limited control resources. Considering the progress of medical technology, the targeted treatment for patients will be gradually developed, so we will consider the targeted treatment for patients as a control parameter in the system and discuss its optimal control problem. In addition, we analyze the optimal control of the system by using the Hamilton equation and the adjoint equation. In the proof, we may encounter the following problems:

- How to determine the basic regeneration number of the system. In the presence of the diffusion term, it is necessary to select an appropriate method to represent the basic reproduction number $\Re _{0}$, which is an important prerequisite for discussing the dynamic behavior of the system by using $\Re _{0}$ as the threshold value.
- Can the existence of optimal control be obtained? Because of the existence of diffusion terms, it is difficult to define the adjoint equation and the Hamiltonian function of the control system. At the same time, there are some requirements for the selection of parameters in the numerical simulation.

In view of the above problems, this article is organized as follows. In Sect. 2, an SIVR model with incomplete immunity and spatial heterogeneity is established. Furthermore, the well-posedness of the model is derived, meanwhile, the global existence and global attractiveness of the solution are proved. Section 3 is devoted to studying the threshold dynamic behavior of the system. The extinction or persistence of diseases is analyzed by using the basic reproductive number as the threshold. In Sect. 4, the optimal control of the control system is analyzed by taking the treatment for the patient as the control parameter in the system. Meanwhile, the optimal control problem is discussed by using the Hamiltonian function and adjoint equation. Finally, in Sect. 5, the corresponding results are verified by numerical simulation.

## Model formulation and well-posedness

In this paper, the spatial heterogeneity of the spread of infectious diseases and spatial diffusion is considered. In addition, for vaccines, we consider vaccination rates in susceptible individuals and the effectiveness of the vaccine. Based on model (1.1), we can establish the following epidemic model of SVIR with incomplete immunity. The meanings of parameters in the system (2.1) are shown in Table 1.

2.1 $$ \textstyle\begin{cases} \frac{\partial S}{\partial t}=D_{1}\Delta S+\Lambda (x)-r(x)S-(1-r(x)) \beta (x)SI-d_{1}(x)S, \\ \frac{\partial I}{\partial t}=D_{2}\Delta I+(1-r(x))\beta (x)SI+(1- \eta (x))\frac{\alpha (x)VI}{K(x)+I}-(\gamma (x)+d_{2}(x))I, \\ \frac{\partial V}{\partial t}=D_{3}\Delta V+r(x)S-(1-\eta (x)) \frac{\alpha (x)VI}{K(x)+I}-(\eta (x)+d_{3}(x))V, \\ \frac{\partial R}{\partial t}=D_{4}\Delta R+\gamma (x)I+\eta (x)V-d_{4}(x)R. \end{cases} $$

**Table 1:** Description of parameters of the model

Parameter	Biological implication
$D_{i}$ (*i* = 1,2,3,4)	Diffusion coefficient in susceptibility, infection, vaccination, recovery path
Λ(*x*)	Recruitment rate of the susceptible host
*r*(*x*)	Vaccination coverage rates of susceptible persons
*α*(*x*)	Transmission between infected and vaccinated hosts
*β*(*x*)	Transmission between infected and susceptible hosts
$d_{i}(x)$ (*i* = 1,2,3,4)	Mortality of susceptible, infected, vaccinated, and recovered hosts
*γ*(*x*)	Recovery rate of infected persons
*K*(*x*)	Half-saturation concentration
*η*(*x*)	Effectiveness of vaccine

### Remark 1

We considered that there is a vaccine coverage rate $r(x)$ for susceptible path *S*, and unvaccinated susceptible persons will be injected into the infected path with a transmission rate $\beta (x)$. The susceptible person who has been vaccinated enters the vaccinated compartment, suppose the effectiveness rate of the vaccine to be $\eta (x)$, and if the vaccine fails, the vaccinator will also be injected into the infected path since the inoculated host has some resistance to the virus after being vaccinated. Thus this propagation process is assumed to obey a half-saturation rate $\frac{(1-\eta (x))\alpha (x)VI}{K(x)+I}$ and $\beta (x)>\alpha (x)$.

In addition, because $R(t)$ does not appear in the first three equations of (2.1), we denote system (2.1) as

2.2 $$ \textstyle\begin{cases} \frac{\partial S}{\partial t}=D_{1}\Delta S+\Lambda (x)-r(x)S-(1-r(x)) \beta (x)SI-d_{1}(x)S, \\ \frac{\partial I}{\partial t}=D_{2}\Delta I+(1-r(x))\beta (x)SI+(1- \eta (x))\frac{\alpha (x)VI}{K(x)+I} \\ \hphantom{\frac{\partial I}{\partial t}={}}{}-(\gamma (x)+d_{2}(x))I,\quad x\in \Omega ,t\in [0,\infty ), \\ \frac{\partial V}{\partial t}=D_{3}\Delta V+r(x)S-(1-\eta (x)) \frac{\alpha (x)VI}{K(x)+I}-(\eta (x)+d_{3}(x))V, \end{cases} $$

with the initial value and boundary conditions

$$ \textstyle\begin{cases} \frac{\partial S}{\partial \nu}=\frac{\partial I}{\partial \nu}= \frac{\partial V}{\partial \nu}=0,\quad x\in \partial \Omega ,t>0, \\ (S,I,V)(\cdot ,0)=(S_{0},I_{0},V_{0})(x)>0,\quad x\in \Omega . \end{cases} $$

It is sufficient to determine the dynamics of (2.1). Here, Ω is a smooth bounded region in $\mathbb{R}^{n}$. Define a Banach space $\mathbb{X}:=C(\overline{\Omega},\mathbb{R}^{3})$ with the supremum norm $\|\cdot \|$ and $\mathbb{X}\mathbbm{^{+}}=C(\overline{\Omega},\mathbb{R}^{3}_{+})$. Next, we mainly analyze the dynamic behavior of system (2.2).

Let $\mathcal{T}_{i}:C(\overline{\Omega},\mathbb{R})\to C( \overline{\Omega},\mathbb{R})$ ($i=1,2,3$) be the $C_{0}$-semigroup associated with $D_{1}\Delta -(r(x)+d_{1}(x))$, $D_{2}\Delta -(\gamma (x)+d_{2}(x))$, $D_{3} \Delta -(\eta (x)+d_{3}(x))$. For any $\varphi \in C(\Omega ,\mathbb{R})$, $\mathcal{T}_{i}$ is given by the following formula:

$$\begin{aligned}& \bigl(\mathcal{T}_{1}(t)\varphi \bigr) (x)=e^{-d_{1}(x)t} \int _{\Omega}\Gamma _{1}(t,x,y) \varphi (y)\,dy ,\\& \bigl(\mathcal{T}_{2}(t)\varphi \bigr) (x)=e^{-(\gamma (x)+d_{2}(x))t} \int _{ \Omega}\Gamma _{2}(t,x,y)\varphi (y)\,dy , \end{aligned}$$

and

$$ \bigl(\mathcal{T}_{3}(t)\varphi \bigr) (x)=e^{-(\eta (x)+d_{3}(x))t} \int _{ \Omega}\Gamma _{3}(t,x,y)\varphi (y)\,dy , $$

where $\Gamma _{i}$ ($i=1,2,3$) is the Green function associated with the operator $\frac{\partial n}{\partial t}=\Delta n$ in Ω̅ subject to the boundary condition. With [14, Section 7], $\mathcal{T}=(\mathcal{T}_{1},\mathcal{T}_{2},\mathcal{T}_{3})$ are compact and strongly positive. Set

2.3 $$ \textstyle\begin{cases} F_{1}(\phi )(x)=\Lambda (x)-(1-r(x))\beta (x)\phi _{1}(x)\phi _{2}(x), \\ F_{2}(\phi )(x)=(1-r(x))\beta (x)\phi _{1}(x)\phi _{2}(x)+(1-\eta (x)) \frac{\alpha (x)\phi _{3}(x)\phi _{2}(x)}{K(x)+\phi _{2}(x)}, \\ F_{3}(\phi )(x)=r(x)\phi _{1}(x)-(1-\eta (x)) \frac{\alpha (x)\phi _{3}(x)\phi _{2}(x)}{K(x)+\phi _{2}(x)}. \end{cases} $$

Then we can rewrite (2.2) as the following integral equation:

$$ P(t)=\mathcal{T}\phi + \int _{0}^{t}\mathcal{T}(t-s)\mathcal{F} \bigl(P(s)\bigr)\,ds $$

for $\mathcal{F}=(F_{1},F_{2},F_{3})$ and $\phi =(\phi _{1},\phi _{2},\phi _{3})(x)=(S_{0},I_{0},V_{0})(x)$.

For a positive and continuous function $\zeta (x)$ on Ω̅, define

$$ \zeta _{+}=\max \bigl\{ \zeta (x)\bigr\} , \qquad \zeta _{-}= \min \bigl\{ \zeta (x)\bigr\} . $$

Thus, for the local solution of (2.2), we have the following.

### Lemma 2.1

*System* (2.2) *with any initial value* *ϕ* *for* $t\in [0,\tau _{\mathrm{trans}})$ (*where* $\tau _{\mathrm{trans}}\leq \infty $) *has a unique solution* $P(x,t,\phi )=(S(x,t),I(x,t),V(x,t))$ *with* $P(\cdot ,0,\phi )=\phi $. *Moreover*, $P(x,t,\phi )=(S(x,t),I(x,t),V(x,t))$ *is a classical solution*.

The proof is shown in Appendix A.

In the remainder of this section we will prove the global existence and boundedness of the solution. Consider the following equation:

2.4 $$ \textstyle\begin{cases} \frac{\partial \omega}{\partial t}=D\Delta \omega +\Lambda (\cdot )- \mu (\cdot )\omega , \\ \frac{\partial \omega}{\partial \nu}=0, \end{cases} $$

where $D>0$ and $\Lambda (\cdot )$, $\mu (\cdot )$ are positive and continuous functions on Ω. Thus we have the following.

### Lemma 2.2

([9, Lemma 1])

*System* (2.4) *admits a positive steady state* $\omega _{0}$ *which is unique and asymptotically stable*. *Furthermore*, *if* $\Lambda (x)\equiv \Lambda $, $\mu (x)\equiv \mu $ *are constants*, *thus* $\omega _{0}=\frac{\Lambda}{\mu}$.

The following theorem proves the boundedness of the model.

### Theorem 2.1

*For* $x\in \Omega $, $t\in [0,\infty )$, *the solution of system* (2.2) *satisfies*

$$ \Phi (t)\phi =P(\cdot ,t,\phi )=\bigl(S(\cdot ,t,\phi ),I(\cdot ,t,\phi ),V( \cdot ,t,\phi )\bigr),\quad \forall x\in \overline{\Omega}, t\in [0,\infty ),$$

*where* $\Phi (t)$ *is the semiflow associated with the solution*. *Moreover*, $\Phi (t)$ *is ultimately bounded*.

The proof is shown in Appendix B.

From what has been discussed above, we can get the following results.

### Lemma 2.3

*The semiflow* $\Phi (t):\mathbb{X}^{+}\to \mathbb{X}^{+}$ *admits a compact and global attractor*.

### Proof

With Theorem 2.1 we ensure the ultimate boundedness of system (2.2). Notice that the equation of (2.2) has the diffusion term, which ensures that $\Phi (t)$ is compact. With the direct consequence in [15, Theorem 2.4.6], we can complete the proof. □

In Sect. 3, we analyze the threshold dynamics of system (2.2). The global stability of disease-free equilibrium (DFE) and endemic equilibrium (EE) is analyzed by establishing the relationship between the basic reproduction number $\Re _{0}$ and the principal eigenvalue.

## Threshold dynamics

### Basic reproduction number

In this section, applying the methods in [16, Sect. 3], we give the basic reproduction number $\Re _{0}$ of (2.2). It is easy to see that (2.2) admits a disease-free equilibrium (DFE) $P_{0}=(S^{0},0,V^{0})$, where $S^{0}$, $V^{0}$ satisfy

3.1 $$ \textstyle\begin{cases} \frac{\partial S}{\partial t}=D_{1}\Delta S+\Lambda (\cdot )-(r( \cdot )+d_{1}(\cdot ))S, \\ \frac{\partial V}{\partial t}=D_{3}\Delta V+r(\cdot )S-(\eta (\cdot )+d_{3}( \cdot ))V. \end{cases} $$

Linearizing (2.2) at $P_{0}$, we can get the following system:

$$ \textstyle\begin{cases} \frac{\partial S}{\partial t}=D_{1}\Delta (S-S^{0})-(1-r(\cdot )) \beta (\cdot )S^{0}I-(r(\cdot )+d_{1}(\cdot ))(S-S^{0}), \\ \frac{\partial I}{\partial t}=D_{2}\Delta I+(1-r(\cdot ))\beta ( \cdot )S^{0}I+(1-\eta (\cdot )) \frac{\alpha (\cdot )V^{0}I}{K(\cdot )}-(\gamma (\cdot )+d_{2}(\cdot ))I, \\ \frac{\partial V}{\partial t}=D_{3}\Delta (V-V^{0})+r(\cdot )(S-S^{0})-(1- \eta (\cdot ))\frac{\alpha (\cdot )V^{0}I}{K(\cdot )}-(\eta (\cdot )+d_{3}( \cdot ))(V-V^{0}). \end{cases} $$

In order to discuss the basic reproduction number $\Re _{0}$, we will focus on the linearized equation of infected person *I*.

3.2 $$ \textstyle\begin{cases} \frac{\partial I}{\partial t}=D_{2}\Delta I+(1-r(\cdot ))\beta ( \cdot )S^{0}I+(1-\eta (\cdot )) \frac{\alpha (\cdot )V^{0}I}{K(\cdot )}-(\gamma (\cdot )+d_{2}(\cdot ))I, \\ \frac{\partial I}{\partial \nu}=0,\quad x\in \partial \Omega , t>0. \end{cases} $$

Substituting $I(\cdot ,t)=e^{\lambda t}\delta (\cdot )$, we consider the following subsystem:

3.3 $$ \textstyle\begin{cases} D_{2}\Delta \delta + [(1-r(\cdot ))\beta (\cdot )S^{0}+(1-\eta ( \cdot ))\frac{\alpha (\cdot )V^{0}}{K(\cdot )} ]\delta -(\gamma ( \cdot )+d_{2}(\cdot ))\delta =\lambda \delta , \\ \frac{\partial \delta}{\partial \nu}=0. \end{cases} $$

Thus, define

$$ \mathbb{F}=\bigl(1-r(\cdot )\bigr)\beta (\cdot )S^{0}+\bigl(1-\eta ( \cdot )\bigr) \frac{\alpha (\cdot )V^{0}}{K(\cdot )}, \qquad \mathbb{B}=\Delta D_{2}( \cdot )-\bigl(\gamma (\cdot )+d_{2}(\cdot )\bigr), $$

then the next generation operator is defined as $\mathcal{L}=-\mathbb{F}\mathbb{B}^{-1}$. Set $T_{\mathrm{trans}}$ as the $C_{0}$-semigroup associated with $\mathbb{B}$, then

$$ \mathcal{L}\phi (\cdot )= \int _{0}^{\infty}\mathbb{F}(\cdot )T_{\mathrm{trans}}(t) \phi (\cdot )\,dt =\mathbb{F}(\cdot ) \int _{0}^{\infty}(\cdot )T_{\mathrm{trans}}(t) \phi (\cdot )\,dt ,\quad x\in \overline{\Omega}. $$

We can use the variational formula to give the solution of the eigenvalue problem as

$$\begin{aligned} \lambda _{0} =&-\inf \biggl\{ \int _{\Omega} \biggl(D_{2}(\cdot ) \vert \nabla \delta \vert ^{2} \\ &{}- \biggl[\bigl(1-r(\cdot )\bigr)\beta (\cdot )S^{0}+\bigl(1-\eta (\cdot )\bigr) \frac{\alpha (\cdot )V^{0}}{K(\cdot )}-\bigl(\gamma (\cdot )+d_{2}(\cdot )\bigr) \biggr]\delta ^{2} \biggr)\,dx : \\ &\delta \in H^{1}(\Omega ), \int _{\Omega} \delta ^{2}=1 \biggr\} . \end{aligned}$$

By [16, Theorem 3.2], we have

3.4 $$ \Re _{0}=\varrho (\mathcal{L})=\sup _{\delta \in H^{1},\delta \neq 0} \biggl\{ \frac{\int _{\Omega}[(1-r(\cdot ))\beta (\cdot )S^{0}+ \frac{(1-\eta (\cdot ))\alpha (\cdot )V^{0}}{K(\cdot )}]\delta ^{2}\,dx }{\int _{\Omega}(D_{2} \vert \nabla \delta \vert ^{2}+ (\gamma (\cdot )+d_{2}(\cdot ))\delta ^{2})\,dx } \biggr\} , $$

and $\varrho (\mathcal{L})$ is the spectral radius of $\mathcal{L}$.

#### Remark 2

If all parameters are all constants, $S^{0}=\frac{\Lambda}{r+d_{1}}$, $V^{0}= \frac{r\Lambda}{(r+d_{1})(\eta +d_{3})}$, we have

$$ \Re _{0}=\frac{1}{\lambda _{0}}= \biggl( \frac{(1-r)\beta \Lambda}{r+d_{1}}+ \frac{(1-\eta )\alpha r\Lambda}{K(r+d_{1})(\eta +d_{3})} \biggr)\Big/( \gamma +d_{2}). $$

From Remark 2, we can see the relationship between $\Re _{0}$ and parameters. We have the following lemma on the impact of $D_{2}$ with $\Re _{0}$.

#### Lemma 3.1

*For the basic reproduction number* $\Re _{0}$, *we have*:

1. $\Re _{0}=1/\lambda _{0}$;
2. *For* $D_{2}>0$, $\Re _{0}$ *is a positive and strictly monotonic decline function*;
3. $\Re _{0}\to \max \{ \frac{(1-r(\cdot ))\beta (\cdot )S^{0}+\frac{(1-\eta (\cdot )) \alpha (\cdot )V^{0}}{K(\cdot )}}{\gamma (\cdot )+d_{2}(\cdot )} \}$ *for* $D_{2}\to 0$;
4. $\Re _{0}\to \frac{\int _{\Omega}[(1-r(\cdot ))\beta (\cdot )S^{0}+ \frac{(1-\eta (\cdot ))\alpha (\cdot )V^{0}}{K(\cdot )}]\,dx }{|\Omega | (\gamma (\cdot )+d_{2}(\cdot ))}$ *for* $D_{2}(\cdot )\to \infty $;
5. *For* $\int _{\Omega}[(1-r(\cdot ))\beta (\cdot )S^{0}+ \frac{(1-\eta (\cdot ))\alpha (\cdot )V^{0}}{K(\cdot )}]\,dx <|\Omega |( \gamma (\cdot )+d_{2}(\cdot ))$, *there exists* $D^{*}_{2}$ *such that*, *for* $D_{2}< D^{*}_{2}$, $\Re _{0}>1$ *and* $D_{2}>D^{*}_{2}$, $\Re _{0}<1$; *For* $\int _{\Omega}[(1-r(\cdot ))\beta (\cdot )S^{0}+ \frac{(1-\eta (\cdot ))\alpha (\cdot )V^{0}}{K(\cdot )}]\,dx >|\Omega |( \gamma (\cdot )+d_{2}(\cdot ))$, $\Re _{0}>1$ *for all* $D_{2}>0$.

Define the principal eigenvalue of (3.3) as $\lambda _{0}$. Thus, we have the following result.

#### Lemma 3.2

$\Re _{0}-1$ *has the same sign as* $\lambda _{0}$.

The proof is shown in Appendix C.

### Extinction of disease

In this subsection we give the proof of $\Re _{0}<1$, the stability of DFE.

#### Theorem 3.1

*If* $\Re _{0}<1$, *the disease*-*free equilibrium* $P_{0}$ *is globally asymptotically stable*. *Thus*, *for* $x\in \Omega $,

$$ \lim_{t\to \infty}P(\cdot ,t,\phi )=P_{0}(\cdot ). $$

#### Proof

Applying Lemma 3.1, we can infer that for $\Re _{0}<1$ the principal eigenvalue $\lambda _{0}<0$. By the equation of *S*, *V* in (2.2), with continuity, there exist $\upsilon _{1}$ and $t_{1}>0$

$$ S(\cdot ,t)< S^{0}+\upsilon _{1}, \qquad V(\cdot ,t)< V^{0}+\upsilon _{1}. $$

Thus, for $x\in \Omega $, $t\in [t_{1},\infty )$, the eigenvalue problem

3.5 $$ \textstyle\begin{cases} \frac{\partial \xi}{\partial t}=D_{2}\Delta \xi +(1-r(\cdot ))\beta ( \cdot )(S^{0}+\upsilon _{1})\xi +(1-\eta (\cdot )) \frac{\alpha (\cdot )(V^{0}+\upsilon _{1})\xi}{K(\cdot )}-(\gamma ( \cdot )+d_{2}(\cdot ))\xi , \\ \frac{\partial \xi}{\partial \nu}=0, \end{cases} $$

has a principal eigenvalue $\lambda ^{\upsilon _{1}}_{0}<0$. Thus, (3.2) implies

$$ \textstyle\begin{cases} \frac{\partial I}{\partial t}\leq D_{2}\Delta I+(1-r(\cdot ))\beta ( \cdot )(S^{0}+\upsilon _{1})I+(1-\eta (\cdot )) \frac{\alpha (\cdot )(V^{0}+\upsilon _{1})I}{K(\cdot )}-(\gamma ( \cdot )+d_{2}(\cdot ))I, \\ \frac{\partial I}{\partial \nu}=0. \end{cases} $$

By the comparison principle, we can find a positive constant $\vartheta _{1}$ which satisfies

$$ I(\cdot ,t,\phi )\leq \vartheta _{1}e^{\lambda ^{\upsilon _{1}}_{0}(t-t_{1})} \xi ^{\upsilon _{1}},\quad t\in [t_{1},\infty ), $$

where $\xi ^{\upsilon _{1}}$ is a strong positive eigenfunction associated with $\lambda ^{\upsilon _{1}}_{0}$. Since $\lambda ^{\upsilon _{1}}_{0}<0$, we directly have

$$ \lim_{t\to \infty}I(\cdot ,t,\phi )=0. $$

Thus the equation of *S*, *V* in (2.2) is asymptotic to (3.1), with asymptotic autonomous semiflow theory in [17, Corollary 4.3], such that

$$ \lim_{t\to \infty}S(\cdot ,t,\phi )=S^{0},\qquad \lim _{t\to \infty}V( \cdot ,t,\phi )=V^{0}. $$

This completes the proof. □

### Disease persistence

In this section, we prove the global asymptotic stability of the endemic equilibrium in the case of $\Re _{0}>1$. First, using [10, Lemma 4.1], we get the following conclusion, which ensures that (2.2) has a positive epidemic equilibrium.

#### Lemma 3.3

*Let* $S(x,t)$, $I(x,t)$, $V(x,t)$ *be the solution of* (2.2) *with the initial value* *ϕ*. *If there exists a positive* $t^{*}$ *such that* $I(\cdot ,t^{*},\phi )>0$, *thus for any* $t\geq t^{*}$, $I(\cdot ,t,\phi )>0$. *Moreover*, *for* *S*, *V*,

$$ \liminf_{t\to \infty}S(\cdot ,t,\phi )\geq \frac{\Lambda _{*}}{r^{*}+(1-r_{*})\beta ^{*}I^{*}+d^{*}_{1}} $$

*and*

$$ \liminf_{t\to \infty}V(\cdot ,t,\phi )\geq \frac{r_{*}\Lambda _{*}}{[r^{*}+(1-r_{*})\beta ^{*}I^{*}+d^{*}_{1}][(1-\eta _{*})\alpha ^{*}I^{*}+(\eta ^{*}+d^{*}_{3})]}, $$

*where* $h^{*}=\sup_{x\in \overline{\Omega}}h(\cdot )$, $h_{*}=\inf_{x\in \overline{\Omega}}h(\cdot )$.

The proof is shown in Appendix D.

Next, we conclude this section by proving the global stability of the endemic equilibrium.

#### Theorem 3.2

*For* $\Re _{0}>1$, (2.2) *admits at least one positive steady state*, *and we can find positive* *ε* *for any* $\phi \in \mathbb{X}^{+}$ *with* $S_{0},I_{0},V_{0}\not \equiv 0$ *such that*

$$ \liminf_{t\to \infty}P\geq \varepsilon ,\quad P=\bigl(S(\cdot ,t,\phi ),I( \cdot ,t,\phi ),V(\cdot ,t,\phi )\bigr). $$

#### Proof

Define two sets as  and  With Lemma 3.3, for $\phi _{2}\in{\mathbb{H}_{0}}$, we can find that $x\in \Omega $, $\forall t\geq 0$, which implies $I(\cdot ,t,I^{0})>0$ and . Set  here $\omega (\phi )$ is an omega limit set.

**Claim 1.** $\omega (\phi )=\{P_{0}\}$.

With , we know that , $\forall t \geq 0$, thus we have $I(\cdot , t;\phi )\equiv 0$. Thus we can find that (2.2) is asymptotic to

3.6 $$ \textstyle\begin{cases} \frac{\partial S}{\partial t}=D_{1}\Delta S+\Lambda (\cdot )-r( \cdot )S-d_{1}(\cdot )S, \\ \frac{\partial V}{\partial t}=D_{3}\Delta V+r(\cdot )S-(\eta (\cdot )+d_{3}( \cdot ))V. \end{cases} $$

Then, with Lemma 2.2, on $x\in \overline{\Omega}$, *S*, *I* satisfy $\lim_{t\rightarrow \infty}S(\cdot , t;\phi )=S^{0}(\cdot )$ and $\lim_{t\rightarrow \infty}V(\cdot , t;\phi )=V^{0}(\cdot )$, uniformly. Hence $\omega (\phi )=\{P_{0}\}$, $\forall \phi \in M_{\partial}$.

**Claim 2.** $P_{0}$ satisfies

$$ \limsup_{t\rightarrow \infty} \bigl\Vert \Phi (t)\phi -P_{0} \bigr\Vert \geq \sigma _{0},\quad \forall \phi \in \mathbb{H}_{0}, $$

where $\sigma _{0}>0=\min \{\sigma ^{*}_{0},\delta ^{*}_{0}\}$ is a positive constant, here $\sigma ^{*}_{0}$, $\delta ^{*}_{0}$ will be defined in what follows.

First, by using [16, Lemma 2.2], there exists positive $\sigma ^{*}_{0}>0$ sufficiently small, the following eigenvalue problem

3.7 $$ \textstyle\begin{cases} \frac{\partial \hat{\psi}_{2}}{\partial t}=D_{2}\Delta \hat{\psi}_{2}+(1-r( \cdot ))\beta (\cdot )(S^{0}-\sigma ^{*}_{0})\hat{\psi}_{2}+(1-\eta ( \cdot ))\alpha (\cdot )(V^{0}-\sigma ^{*}_{0})\hat{\psi}_{2}( \frac{1}{K(\cdot )}-\sigma ^{*}_{0}) \\ \hphantom{\frac{\partial \hat{\psi}_{2}}{\partial t}={}}{}-(\gamma (\cdot )+d_{2}(\cdot )) \hat{\psi}_{2}, \\ \frac{\partial \hat{\psi}_{2}}{\partial \nu}=0 \end{cases} $$

admits a principal eigenvalue $\lambda ^{\sigma _{0}}_{0}$. To deal with nonlinear terms in the system, we can choose $\delta ^{*}_{0}$ with

$$ \frac{1}{K(\cdot )+I}>\frac{1}{K(\cdot )}-\sigma ^{*}_{0},\quad I< \delta ^{*}_{0}. $$

Then, to the contrary, assume that there is positive $\sigma _{0}>0$ such that, for $\phi \in \mathbb{H}_{0}$,

$$ \limsup_{t\rightarrow \infty} \bigl\Vert \Phi (t)\phi -P_{0} \bigr\Vert < \sigma _{0}. $$

It follows that there exists $t_{3}>0$ for $x\in \overline{\Omega}$ which satisfies

$$ S^{0}(\cdot )-\sigma _{0}< S(\cdot ,t,\phi ),\qquad I(\cdot ,t, \phi ) < \sigma _{0}, \qquad V^{0}-\sigma _{0}< V( \cdot ,t,\phi ) ,\quad \forall t\geq t_{2}. $$

Define $\hat{I}(\cdot ,t)$ for $x\in \Omega $, $t\in [0,\infty )$ which satisfies

3.8 $$ \textstyle\begin{cases} \frac{\partial \hat{I}}{\partial t}=D_{2}\Delta \hat{I}+(1-r(\cdot )) \beta (\cdot )(S^{0}-\sigma _{0})\hat{I}+(1-\eta (\cdot ))\alpha ( \cdot )(V^{0}-\sigma _{0})\hat{I}(\frac{1}{K(\cdot )}-\sigma _{0}) \\ \hphantom{\frac{\partial \hat{I}}{\partial t}={}}{}-( \gamma (\cdot )+d_{2}(\cdot ))\hat{I}, \\ \frac{\partial \hat{I}}{\partial \nu}=0. \end{cases} $$

Thus, for $\varsigma >0$, $\hat{I}(\cdot ,t)=\varsigma \hat{\psi}_{2{\sigma _{0}}}e^{(t-t_{3}) \lambda ^{\sigma _{0}}_{0}}$ is the unique solution of (3.7). ($\hat{\psi}_{2{\sigma _{0}}}$ is the strong positive eigenfunction corresponding to $\lambda ^{\sigma _{0}}_{0}$). For , with Lemma 3.3, it follows that $I(\cdot ,t,\phi )>0$. From the definition of the upper solution, for the solution of (3.8) and $I(\cdot ,t,\phi )$, we can find that

$$ I(\cdot ,t,\phi )\geq \hat{I}(\cdot ,t),\quad \overline{\Omega}\times [t_{3}, \infty ). $$

By the comparison principle, we can find a small positive constant *ς* which satisfies

$$ I(\cdot ,t;\phi )\geq \varsigma \hat{\psi}_{2{\sigma _{0}}}e^{(t-t_{3}) \lambda ^{\sigma _{0}}_{0}},\quad t \geq t_{3}. $$

Thus, for $\Re _{0}>1$, with Lemma 3.2, which implies $\lambda ^{\sigma _{0}}_{0}>0$, then $I(\cdot ,t,\phi )\rightarrow \infty $ when $t\rightarrow \infty $. It means that $I(\cdot ,t,\phi )$ is unbounded, which contradicts the previous proof. This completes the proof.

Here we give the definition of a distance function in the semiflow $\Phi (t): \mathbb{X}^{+}\to \mathbb{X}^{+}$ as follows:

$$ c(\phi ):=\min_{x\in \overline{\Omega}}\bigl\{ \phi _{2}(\cdot ) \bigr\} ,\quad \forall \phi \in \mathbb{X}^{+}, $$

where $c(\cdot ): \mathbb{X}^{+}\to [0,\infty )$. Hence, applying [10, Theorem 4.1], for any $\tau _{1} > 0$, we have  and  By Lemma 3.3, we can set

$$ \varepsilon _{2}=\min \biggl\{ \frac{\Lambda _{*}}{r^{*}+(1-r_{*})\beta ^{*}I^{*}+d^{*}_{1}}, \frac{r_{*}\Lambda _{*}}{[r^{*}+(1-r_{*})\beta ^{*}I^{*}+d^{*}_{1}][(1-\eta _{*})\alpha ^{*}I^{*}+(\eta ^{*}+d^{*}_{3})]} \biggr\} , $$

thus $\liminf_{t\to \infty}S(\cdot ,t,\phi ),\liminf_{t\to \infty}V( \cdot ,t,\phi )\geq \varepsilon _{2}$. Set $\varepsilon =\min \{\varepsilon _{1},\varepsilon _{2}\}$, we can get the result that the endemic equilibrium is uniformly persistent. Therefore, by [18, Theorem 4.7], for (2.2), there is at least one positive steady state of on . □

## Optimal control

In the previous chapters, we have focused on the disease-free equilibrium and the infectious equilibrium of infectious diseases. But if there is a sudden outbreak, we need to control the impact of the disease at a lower level as far as possible, that is, to control the number of infected people. In addition to calling for vaccinations, governments often spend more money on treatment. The mathematical language to describe this method is the optimal control problem. The main aim of this section is to develop effective strategies for controlling the spread of infectious diseases. We hope that the number of infected people does not exceed the number of susceptible and effective vaccinators.

In this section, we introduce the control strategy to (2.2) and analyze its properties. For convenience, we rewrite $\Lambda (x)$ as Λ, the same for other parameters. To complete our research, we analyze the control variables of the model (2.2). Therefore, the control variables are given as follows.

With the development of medical technology, infected patients can be treated better. Therefore, define $u=u(x,t)$ represents the medical intervention for infected patients. Considering that medical resources are limited, we use $\frac{cuI}{1+\omega I}$ for specific. Here, *c* is the cure rate and *ω* denotes the saturation constant.

From this, we give the control system of (2.2) as follows:

4.1 $$ \textstyle\begin{cases} \frac{\partial S}{\partial t}=D_{1}\Delta S+\Lambda -rS-(1-r)\beta SI-d_{1}S, \\ \frac{\partial I}{\partial t}=D_{2}\Delta I+(1-r)\beta SI+(1-\eta ) \frac{\alpha VI}{K+I}-(\gamma +d_{2})I-\frac{cuI}{1+\omega I},\quad x\in \Omega ,t\in [0,\infty ), \\ \frac{\partial V}{\partial t}=D_{3}\Delta V+rS-(1-\eta ) \frac{\alpha VI}{K+I}-(\eta +d_{3})V, \end{cases} $$

with the boundary condition

$$ \frac{\partial S}{\partial \nu}=\frac{\partial I}{\partial \nu}= \frac{\partial V}{\partial \nu}=0,\quad (x,t)\in \Sigma =(0,T)\times \partial \Omega , $$

here *u* is measurable, other parameters are the same as in (2.2).

Define an objective function

$$ J\bigl(u(x,t)\bigr)= \int _{0}^{T} \int _{\Omega }L\bigl(I(x,t);u(x,t)\bigr)\,dx\, dt , $$

where $u(x,t)\in \mathscr{U}(\Omega \times [0,T])=\{0\leq u(x,t)\leq 1\}$ and $L=A_{1}I(x,t)+\frac{1}{2}A_{2}u^{2}(x,t)$. Assume that the control set $\mathscr{U}(\Omega \times [0,T])$ is convex, $A_{1}$, $A_{2}$ are weight of each item. This objective function describes our goal to control the problem: to reduce the number of susceptible and infected people with minimal intervention costs. The value function is defined as

$$ V\bigl(0,\phi (\cdot ,0)\bigr)=\min_{u(x,t)\in \mathscr{U}(\Omega \times [0,T])}J\bigl(0, \phi ( \cdot ,0);u(\cdot ,t)\bigr). $$

Define a Hilbert space $H=L^{2}(\Omega ^{2})$ and $S^{0}_{c}, I^{0}_{c}, V^{0}_{c}>0$ as the initial value of (4.1), which satisfies (IOC: Initial value of Optimal Control)

$$ (\mathrm{IOC}) \quad S^{0}_{c}, I^{0}_{c} \in H^{2}(\Omega ), \qquad \partial S^{0}_{c}/ \partial \nu =0,\qquad \partial I^{0}_{c}/\partial \nu =0,\qquad \partial V^{0}_{c}/ \partial \nu =0. $$

Let $f^{c}=(f_{1}(t,S,I,V), f_{2}(t,S,I,V), f_{3}(t,S,I,V))$, where

$$ \textstyle\begin{cases} f_{1}(t,S,I,V)=\Lambda -rS-(1-r)\beta SI-d_{1}S, \\ f_{2}(t,S,I,V)=(1-r)\beta SI+(1-\eta )\frac{\alpha VI}{K+I}-(\gamma +d_{2})I- \frac{cuI}{1+\omega I}, \\ f_{3}(t,S,I,V)=rS-(1-\eta )\frac{\alpha VI}{K+I}-(\eta +d_{3})V. \end{cases} $$

With the method in [19, 20], we have the following lemma.

### Lemma 4.1

*For the initial value* $S^{0}_{c}$, $I^{0}_{c}$, $V^{0}_{c}$, *system* (4.1) *admits a unique strong solution* $(S,I,V)\in W^{1,2}(0,T;H)$ *such that* $S,I,V>0$ *and* $(S,I,V)\in L^{2}(0,T;H^{2}(\Omega ))\cap L^{\infty}(0,T;H^{1}( \Omega ))\cap L^{\infty}(\mathscr{U})$. *Furthermore*, *there exists a positive constant* *C* *independent of* *u*, *for any* $t\in [0,T]$,

$$ \begin{aligned} & \biggl\Vert \frac{\partial S}{\partial t} \biggr\Vert _{L^{2}(Q)}+ \Vert S \Vert _{L^{2}(0,T;H^{2}( \Omega ))}+ \bigl\Vert S(t) \bigr\Vert _{H^{1}{(\Omega )}}+ \Vert S \Vert _{L^{\infty}(Q)}\leq C, \\ & \biggl\Vert \frac{\partial I}{\partial t} \biggr\Vert _{L^{2}(Q)}+ \Vert I \Vert _{L^{2}(0,T;H^{2}( \Omega ))}+ \bigl\Vert I(t) \bigr\Vert _{H^{1}{(\Omega )}}+ \Vert I \Vert _{L^{\infty}(Q)}\leq C, \\ & \biggl\Vert \frac{\partial V}{\partial t} \biggr\Vert _{L^{2}(Q)}+ \Vert V \Vert _{L^{2}(0,T;H^{2}( \Omega ))}+ \bigl\Vert V(t) \bigr\Vert _{H^{1}{(\Omega )}}+ \Vert V \Vert _{L^{\infty}(Q)}\leq C, \end{aligned} $$

*where* $Q=\Omega \times [0,T]$.

Lemma 4.1 ensures the existence and boundedness of the global solution of system (4.1). Next, according to [21], we can analyze the existence of optimal control of system (4.1).

### Theorem 4.1

*Let the initial value be defined in* (*IOC*). *Then there exists an optimal solution* $P'=(S',I',V')$ *of the control system* (4.1) *corresponding to optimal control* $u'$.

### Proof

From the boundedness we have proved in Sect. 3, we can infer that $p=\inf{J(u(x,t))}$ is finite. Thus, for $P_{n}=(S_{n},I_{n},V_{n})$ and $u_{n}\in \mathscr{U}$,we can find a sequence $(P_{n},u_{n})$ that is the solution to the following subsystem:

4.2 $$ \textstyle\begin{cases} \frac{\partial S_{n}}{\partial t}=D_{1}\Delta S_{n}+\Lambda -rS_{n}-(1-r) \beta S_{n}I_{n}-d_{1}S_{n}, \\ \frac{\partial I_{n}}{\partial t}=D_{2}\Delta I_{n}+(1-r)\beta S_{n}I_{n}+(1- \eta )\frac{\alpha V_{n}I_{n}}{K+I_{n}}-(\gamma +d_{2})I_{n} \\ \hphantom{\frac{\partial I_{n}}{\partial t}={}}{}- \frac{cuI_{n}}{1+\omega I_{n}},\quad x\in \Omega ,t\in [0,T], \\ \frac{\partial V_{n}}{\partial t}=D_{3}\Delta V_{n}+rS_{n}-(1-\eta ) \frac{\alpha V_{n}I_{n}}{K+I_{n}}-(\eta +d_{3})V_{n}, \end{cases} $$

with the initial condition

$$ S_{n}(0,t)=S_{0}(x),\qquad I_{n}(0,t)=I_{0}(x),\qquad V_{n}(0,t)=V_{0}(x) $$

and the boundary condition

$$ \frac{\partial S_{n}}{\partial \nu}= \frac{\partial I_{n}}{\partial \nu}= \frac{\partial V_{n}}{\partial \nu}=0,\quad (x,t)\in \Sigma , $$

such that

$$ p\leq J\bigl(u(x,t)\bigr)\leq p+\frac{1}{n}, \quad \forall n\geq 1. $$

With Lemma 4.1, for system (4.2), we can infer that

4.3 $$ \begin{aligned} & \biggl\Vert \frac{\partial S_{n}}{\partial t} \biggr\Vert _{L^{2}(Q)}+ \Vert S_{n} \Vert _{L^{2}(0,T;H^{2}( \Omega ))}+ \bigl\Vert S_{n}(t) \bigr\Vert _{H^{1}{(\Omega )}}+ \Vert S_{n} \Vert _{L^{\infty}(Q)} \leq C, \\ & \biggl\Vert \frac{\partial I_{n}}{\partial t} \biggr\Vert _{L^{2}(Q)}+ \Vert I_{n} \Vert _{L^{2}(0,T;H^{2}( \Omega ))}+ \bigl\Vert I_{n}(t) \bigr\Vert _{H^{1}{(\Omega )}}+ \Vert I_{n} \Vert _{L^{\infty}(Q)} \leq C, \\ & \biggl\Vert \frac{\partial V_{n}}{\partial t} \biggr\Vert _{L^{2}(Q)}+ \Vert V_{n} \Vert _{L^{2}(0,T;H^{2}( \Omega ))}+ \bigl\Vert V_{n}(t) \bigr\Vert _{H^{1}{(\Omega )}}+ \Vert V_{n} \Vert _{L^{\infty}(Q)} \leq C. \end{aligned} $$

Since $H^{1}(\Omega )$ is compactly imbedded in $L^{2}(\Omega )$, we can also get the compactness of $S_{n}$, $I_{n}$, $V_{n}$ and $\frac{\partial S_{n}}{\partial t}$, $\frac{\partial I_{n}}{\partial t}$, $\frac{\partial V_{n}}{\partial t}$. Here, by using the Arzela–Ascoli theorem [22], for the compactness we proved in Sect. 2,

$$ S_{n}\to S', \qquad I_{n}\to I',\qquad V_{n}\to V', $$

uniformly in $L^{2}(\Omega )$ with respect to a subsequence denoted by $P_{n}$. In addition, with the weak convergence of $\Delta S_{n}$, $\Delta I_{n}$, $\Delta V_{n}$ (with the boundedness in system (4.2)), we have

$$ \Delta S_{n}\to \Delta S',\qquad \Delta I_{n} \to \Delta I', \qquad \Delta V_{n} \to \Delta V' $$

weakly in $L^{2}(Q)$. With (4.3), we have for $P'=(S',I',V')$ and $P_{n}=(S_{n}, I_{n}, V_{n})$

$$\begin{aligned}& \frac{\partial P_{n}}{\partial t}\to \frac{\partial P'}{\partial t} \quad \text{weakly in } L^{2}(Q),\\& P_{n}\to P' \quad \text{weakly star in } L^{\infty}\bigl([0,T]; H^{1}(\Omega )\bigr),\\& P_{n}\to P' \quad \text{weakly in } L^{2}\bigl([0,T]; H^{1}(\Omega )\bigr). \end{aligned}$$

Next, we focus on the second equation of (4.2). By direct calculation we have

$$ \begin{aligned} &(1-r)\beta S_{n}I_{n}+(1-\eta )\frac{\alpha V_{n}I_{n}}{K+I_{n}}- \biggl[(1-r)\beta S'I'+(1- \eta )\frac{\alpha V'I'}{K+I'} \biggr] \\ &\quad =(1-r) \beta \bigl(S_{n}I_{n}-S'I' \bigr) +(1-\eta )\frac{\alpha V_{n}I_{n}}{(K+I_{n})(K+I')}\bigl[\bigl(K+I' \bigr)I_{n}\bigl(S_{n}-S' \bigr)+KS'\bigl(I_{n}-I'\bigr)\bigr]. \end{aligned} $$

Thus

$$ (1-r)\beta S_{n}I_{n}+(1-\eta )\frac{\alpha V_{n}I_{n}}{K+I_{n}}\to (1-r) \beta S'I'+(1-\eta )\frac{\alpha V'I'}{K+I'}. $$

Similarly, we can discuss the first and third equation of (4.3). For the subsequence ${u_{n}}$, $u_{n}\to u'$ weakly in $L^{2}(Q)$. With the convexity and closeness of $\mathscr{U}$, hence $u'\in \mathscr{U}$, we have

$$ \frac{cu_{n}I_{n}}{1+\omega I_{n}}\to \frac{cu'I'}{1+\omega I'}. $$

With the analysis above, we can give the conclusion that for $n\to \infty $, $P'=(S',I',V')$ is the optimal solution associated with the optimal function $u'$ of system (4.1), which completes the proof. □

The Hamiltonian function of the control system is given as follows:

4.4 $$ \begin{aligned} &H^{\star}(x,t,S,I,V,u,p) \\ &\quad = A_{1}I(x,t)+\frac{1}{2}A_{2}u^{2}(x,t)+p_{1} \bigl(D_{1} \Delta S+\Lambda -rS-(1-r)\beta SI-d_{1}S \bigr) \\ &\qquad {}+p_{2}\biggl(D_{2}\Delta I+(1-r) \beta SI +(1-\eta )\frac{\alpha VI}{K+I}-(\gamma +d_{2})I- \frac{cuI}{1+\omega I}\biggr) \\ &\qquad {}+p_{3}\biggl(D_{3}\Delta V+rS-(1-\eta ) \frac{\alpha VI}{K+I}-(\eta +d_{3})V\biggr). \end{aligned} $$

Next we give the adjoint equation for the control system (4.1)

4.5 $$ \textstyle\begin{cases} \frac{\partial p_{1}(x,t)}{\partial t} =- \frac{\partial H^{\star}}{\partial S}=[r+(1-r)\beta I+d_{1}]p_{1}-D_{1} \Delta p_{1}-(1-r)\beta Ip_{2}-rp_{3}, \\ \frac{\partial p_{2}(x,t)}{\partial t} =- \frac{\partial H^{\star}}{\partial I} \\ \hphantom{\frac{\partial p_{2}(x,t)}{\partial t}}=(1-r)\beta Sp_{1}+ [\gamma +d_{2} \frac{cu}{(1+\omega I)^{2}}-(1-r)\beta S-(1-\eta ) \frac{\alpha VK}{(K+I)^{2}} ]p_{2} \\ \hphantom{\hphantom{\frac{\partial p_{2}(x,t)}{\partial t}}={}}{} -D_{2}\Delta p_{2}+(1-\eta )\frac{\alpha VK}{(K+I)^{2}}p_{3}-A_{1}, \\ \frac{\partial p_{3}(x,t)}{\partial t} =- \frac{\partial H^{\star}}{\partial V}=-(1-\eta )\frac{\alpha I}{K+I}p_{2}+ [(1-\eta )\frac{\alpha I}{K+I}+\eta +d_{3} ]p_{3}-D_{3}\Delta p_{3}, \\ p_{i}(T) =0,\quad i=1,2,3. \end{cases} $$

Using the method in [23], give the partial derivative of the Hamiltonian function to *u*, substitute it into the optimal control solution $P'$,

$$ \frac{\partial H^{\star}}{\partial u}=A_{2}u- \frac{cp_{2}(x,t)I'}{1+\omega I'}. $$

Let $\frac{\partial H^{\star}}{\partial u}=0$, the optimal control pair $u'$ satisfying the minimum value of the objective function $\min_{u(x,t)\in \mathscr{U}} J(u)$ can be expressed as

$$ u'=\min \biggl\{ \max \biggl\{ \frac{p_{2}(x,t)cI'}{A_{2}(1+\omega I')},0\biggr\} ,1 \biggr\} . $$

## Numerical simulation

In this section, we use numerical simulation to verify the stability of the system and the impact of controls on the development of the disease. The values of each parameter are shown in Table 2.

**Table 2:** Values for the parameters in numerical simulation

Parameter	Data 1	Data 2	Source
$D_{1}$	1.25 × 10^−4^	1.25 × 10^−4^	[10]
$D_{2}$	1.25 × 10^−4^	1.25 × 10^−4^	[10]
$D_{3}$	1.25 × 10^−4^	1.25 × 10^−4^	[10]
Λ	0.4	0.8	Assume
*β*	0.6	0.75	[24]
*η*	0.72	0.62	[25]
*α*	0.5	0.7	[24], Assume
*r*	0.4	0.2	Assume
$d_{1}$	0.1595	0.1595	[26]
$d_{2}$	0.1815	0.2145	Assume
$d_{3}$	0.1595	0.1595	[26]
*γ*	0.9	0.75	[27], Assume
*c*	0.75	0.75	Assume
*ω*	0.5	0.5	Assume

### Stability of equilibrium

In this section, we discuss the stability of the solution of system (2.2). First of all, use the method in [28] to make difference and solve system (2.2). Then, for $\Re _{0}<1$ and $\Re _{0}>1$, take the data in Data 1 and Data 2, respectively. We get the simulation results obtained as follows.

**Figure 1:**
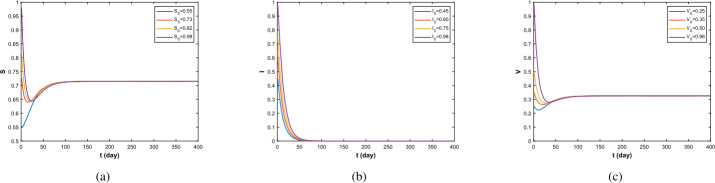
$\Re _{0}=0.322<1$, the density of *S*, *I*, *V*

Firstly, take the value in Data 1. As we can see from Fig. 1, when $\Re _{0}<1$, from Theorem 3.1, the disease-free equilibrium $(S^{0},0,V^{0})$ is asymptotically stable. In fact, as shown in Fig. 1(b), for $t\to \infty $, $I(x,t)$ converges to zero; in addition, $S^{0}=\frac{\Lambda}{\gamma +d_{1}}=0.7149$, $V^{0}= \frac{r\Lambda}{(\gamma +d_{1})(\eta +d_{3})}=0.3252$. This is the same conclusion given by Theorem 3.1.

**Figure 2:**
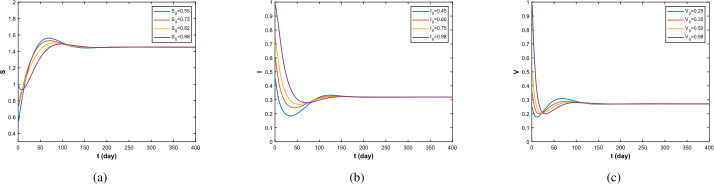
$\Re _{0}=1.721>1$, the density of *S*, *I*, *V*

Then, using Data 2, we get the numerical simulation when $\Re _{0}>1$. With Theorem 3.2, we can get the uniform persistence of disease. Actually, as we can see in Fig. 2(b), with $t\to \infty $, $I(x,t)$ approaches a constant positive value. This is consistent with our proof in Theorem 3.2.

### Influence of control parameters on disease progression

In this section, we discuss the impact of control on a disease. The main idea of this section is that we solve the optimal control problem by applying the iterative method. Then the optimal system is obtained by using the state equation and adjoint equation given in Sect. 4. And by solving the optimal system, the optimal control strategy is obtained. Furthermore, the method in [28] is used to make difference and solve the control system and the adjoint equation. In order to control the susceptible population, the infected population, and the vaccinated population, targeted treatment of patients is taken as control, and the impact of targeted treatment on the susceptible population, the infected population, and the density of the vaccinated population in a long-term state is considered. Finally, through numerical simulation, the actual situation of each path in the original system (2.2) and the control system (4.1) is compared.

First, we define the objective function as follows. Let the objective function corresponding to the control system (4.1) be as follows:

$$ J\bigl(u(x,t)\bigr)= \int _{0}^{T} \int _{\Omega }A_{1}I(x,t)+\frac{1}{2}A_{2}u^{2}(x,t)\,dx\, dt , $$

where $A_{1}=0.4$, $A_{2}=0.5$ [29]. The values of other parameters are the same as in Sect. 5.1. The numerical simulation results are as follows.

**Figure 3:**
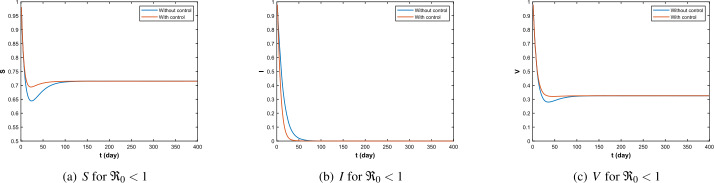
Infectious path *S*, *I*, *V* without and with control ($\Re _{0}<1$)

**Figure 4:**
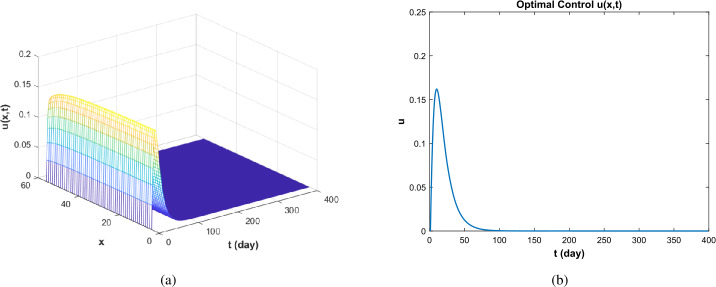
The optimal control when $\Re _{0}<1$

In Fig. 3, for $\Re _{0}<1$, under controlled conditions, the duration of the disease is shorter and the time of extinction is earlier than without control. At the same time, the overall density of susceptible and vaccinated people before reaching the stabilization point was also higher in the controlled condition than without control. In addition, according to Fig. 4, the control intensity reached the maximum in the early stage and gradually decreased with the weakening of the disease scale, reaching zero value when the disease disappeared.

**Figure 5:**
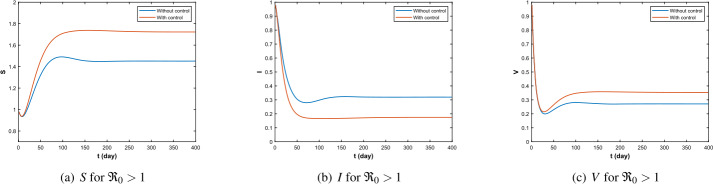
Infectious path *S*, *I*, *V* without and with control ($\Re _{0}>1$)

**Figure 6:**
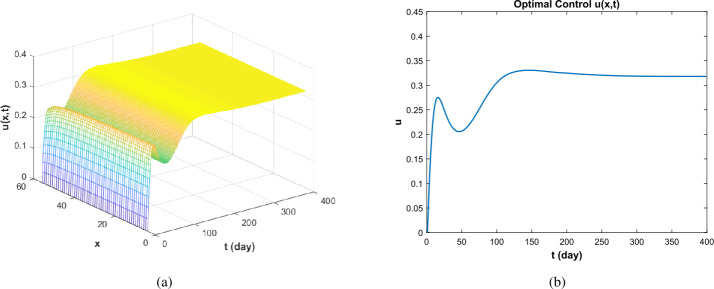
The optimal control when $\Re _{0}>1$

As shown in Fig. 5, for $\Re _{0}>1$, when control exists, the scale of the disease reaches a minimum earlier than without control, and when the disease eventually becomes endemic, the total scale of the disease is lower than without control. In addition, when the disease reaches a stable state, the density of susceptible and vaccinated persons is higher in the controlled condition than without control. Furthermore, by Fig. 6, when the disease is in its initial state of development, control rises, and when the disease reaches equilibrium and becomes endemic, control is maintained at a stable value along with the duration of the disease.

## Conclusion and discussion

**Figure 7:**
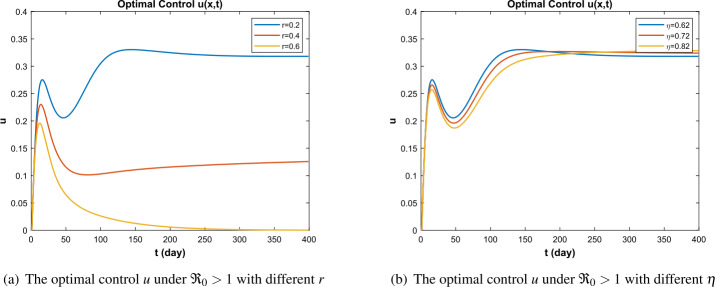
Optimal control under different parameter values

In this paper, a kind of SIVR infectious disease model including vaccine immunity and vaccine effectiveness is considered. The optimal control theory is applied to the study of the model, and the threshold dynamics and optimal control of the model are discussed. Firstly, we prove the well-posedness of the model, which provides a theoretical basis for the following discussion. Secondly, we give the basic reproduction number $\Re _{0}$ to analyze the dynamic behavior of the disease threshold. In addition, the Hamiltonian function and adjoint equation of the optimal control problem is given. Finally, the stability of the system solution is verified by numerical simulation and the number of infections can be reduced as much as possible, while the cost is reduced under the treatment control. In this paper, the parameters are assumed to be accurate; in fact, due to various uncertainties, each parameter may be inaccurate or random. At the same time, according to the changes in the parameters, it can be seen that the vaccination rate and the effective rate of the vaccine also have a certain impact on the control (see Fig. 7(a), (b)). In addition, because the near-optimal control is more flexible, it can adapt to different degrees of model uncertainty. Therefore, in future work, the near-optimal control problem of the epidemic model can be further studied by considering the influence of random parameters, noise, the vaccination rate, and the efficiency rate of the vaccine as the control parameter.

## Data Availability

Data used to support the findings of this work are available from the corresponding author upon request.

## Appendix A: Proof of Lemma 2.1

Define the domain of the linear homogeneous part $\mathcal{T}$ as

$$ D(\mathcal{T})=\biggl\{ \phi :\frac{\partial \phi}{\partial t}=0 \text{ on } \partial \Omega ,\mathcal{T}\phi \in \mathbb{X}\biggr\} . $$

Thus, there exists $h\geq 0$ which satisfies

A.1 $$\begin{aligned} \phi + h\mathcal{F}(\phi )&= \begin{pmatrix} \phi _{1}+h [\Lambda (x)-(1-r(x))\beta (x)\phi _{1}(x)\phi _{2}(x) ] \\ \phi _{2}+h [(1-r(x))\beta (x)\phi _{1}(x)\phi _{2}(x)+(1-\eta (x)) \frac{\alpha (x)\phi _{3}(x)\phi _{2}(x)}{K(x)+\phi _{2}(x)} ] \\ \phi _{3}+h [r(x)\phi _{1}(x)-(1-\eta (x)) \frac{\alpha (x)\phi _{3}(x)\phi _{2}(x)}{K(x)+\phi _{2}(x)} ] \end{pmatrix} \end{aligned}$$

A.2 $$\begin{aligned} &\geq \begin{pmatrix} \phi _{1} [1-h(1-r_{-})\phi _{2}\beta _{+} ] \\ \phi _{2} \\ \phi _{3} [1-h(1-\eta _{-})\phi _{2}\alpha _{+} ] \end{pmatrix}. \end{aligned}$$

We can directly get

$$ \lim_{h\to \infty}\frac{1}{h}\operatorname{dist}\bigl(\phi +h\mathcal{F}, \mathbb{X}^{+}\bigr)=0. $$

With [30, Corollary .4], we can prove the lemma.

## Appendix B: Proof of Theorem 2.1

Define

$$ H(t)= \int _{\Omega}\bigl[S(\cdot ,t)+I(\cdot ,t)+V(\cdot ,t)\bigr]\,dx . $$

Differential with respect on *t*

B.1 $$ \begin{aligned} \frac{\operatorname{d}H(t)}{\operatorname{d}t}&= \int _{\Omega} \Lambda (\cdot )\,dx \\ &\quad {} - \int _{\Omega}\bigl[d_{1}(\cdot )S(\cdot ,t)+\bigl( \gamma ( \cdot )+d_{2}(\cdot )\bigr)I(\cdot ,t)+\bigl(\eta (\cdot )+d_{3}(\cdot )\bigr)V( \cdot ,t)\bigr]\,dx \\ &\leq \Lambda (\cdot ) \vert \Omega \vert -\theta H(t), \end{aligned} $$

where $\theta =\min \{d_{1}(\cdot ),\gamma (\cdot )+d_{2}(\cdot ),\eta ( \cdot )+d_{3}(\cdot )\}$. Hence we have

$$ H(t)\leq H(0)e^{-\theta t}+\frac{\Lambda (\cdot ) \vert \Omega \vert }{\theta}\bigl(1-e^{- \theta t} \bigr)< \infty , $$

thus for some $t\geq t_{1}$ and positive constant *N*, we have $H(t)< N$.

With Lemma 2.2 and first and third equation of (2.2), by the comparison principle, we have, for some $t\geq t_{2}$,

$$ \limsup_{t\to \infty}S(\cdot ,t)\leq \omega _{0},\qquad \limsup_{t\to \infty}V(\cdot ,t)\leq \omega _{1}. $$

Thus, $S(\cdot ,t)$ and $V(\cdot ,t)$ are ultimately bounded. With the above condition, $\mathcal{T}_{2}$ is the $C_{0}$ semigroup associated with $D_{2}\Delta -(\gamma (x\cdot )+d_{2}(\cdot ))$. We can rewrite $\mathcal{T}_{2}$ with a Green function as

$$ \bigl(\mathcal{T}_{2}(t)\phi \bigr) (x)=e^{-(\gamma (\cdot )+d_{2}(\cdot ))t} \int _{\Omega}\Gamma _{2}(t,x,y)\phi (y)\,dy , $$

thus there exists a positive constant $\mathbb{M}$ satisfying $\|\mathcal{T}_{2}\|\leq \mathbb{M}e^{k_{2}t}$, where $k_{2}$ is the principle eigenvalue of $D_{2}\Delta -(\gamma (\cdot )+d_{2}(\cdot ))$. With [31, Theorem 2.4.7], for Green function $\Gamma _{2}$ satisfies

$$ \Gamma _{2}(t,x,y)\leq me^{-(\gamma _{-}+d_{2-})t}, $$

where $m>0$ is a positive constant. By (2.1), we can conclude that, for $t_{m}=\max \{t_{1},t_{2}\}$,

B.2 $$\begin{aligned} I(\cdot ,t)&=\mathcal{T}_{2}I( \cdot ,t_{m})+ \int _{t_{m}}^{t} \mathcal{T}_{2}(t-s)\biggl[ \bigl(1-r(x)\bigr)\beta (x)SI+\bigl(1-\eta (x)\bigr) \frac{\alpha (x)VI}{K(x)+V} \biggr]\,ds \\ &\leq \mathbb{M}e^{k_{2}(t-t_{m})} \bigl\Vert I(\cdot ,t_{m}) \bigr\Vert \\ &\quad {}+ \int _{t_{m}}^{t} \int _{\Omega}\Gamma _{2}(t-s,x,y) \bigl[(1-r_{-})\beta _{+}\omega _{0}+(1- \eta _{-})\alpha _{+}\omega _{1}\bigr]\,dy\, ds \\ &\leq \mathbb{M}e^{k_{2}(t-t_{m})} \bigl\Vert I(\cdot ,t_{m}) \bigr\Vert \\ &\quad {}+ \int _{t_{m}}^{t} me^{-(\gamma _{-}+d_{2-})(t-s)} \biggl\{ \bigl[(1-r_{-})\beta _{+}\omega _{0}+(1- \eta _{-})\alpha _{+}\omega _{1}\bigr] \int _{\Omega }I(s,y)\,dy \biggr\} \,ds \\ &\leq \mathbb{M}e^{k_{2}(t-t_{m})} \bigl\Vert I(\cdot ,t_{m}) \bigr\Vert \\ &\quad {}+ \int _{t_{m}}^{t} me^{-(\gamma _{-}+d_{2-})(t-s)} \bigl\{ \bigl[(1-r_{-})\beta _{+}\omega _{0}+(1- \eta _{-})\alpha _{+}\omega _{1}\bigr]N \bigr\} \,ds \\ &=\mathbb{M}e^{k_{2}(t-t_{m})} \bigl\Vert I(\cdot ,t_{m}) \bigr\Vert + \frac{N_{\mathrm{trans}}(1-e^{-(\gamma _{-}+d_{2-})(t-t_{m})})}{\gamma _{-}+d_{2-}} \\ &\leq \mathbb{M}e^{k_{2}(t-t_{m})} \bigl\Vert I(\cdot ,t_{m}) \bigr\Vert + \frac{N_{\mathrm{trans}}}{\gamma _{-}+d_{2-}}, \end{aligned}$$

where $N_{\mathrm{trans}}=mN[(1-r_{-})\beta _{+}\omega _{0}+(1-\eta _{-})\alpha _{+} \omega _{1}]$. Since $k_{2}$ is the principle eigenvalue associated with $D_{2}\Delta -(\gamma (\cdot )+d_{2}(\cdot ))$, we can get to the conclusion that

$$ \limsup_{t\to \infty} \bigl\Vert I(\cdot ,t) \bigr\Vert \leq \frac{N_{\mathrm{trans}}}{\gamma _{-}+d_{2-}}. $$

Thus $S(\cdot ,t)$, $I(\cdot ,t)$, $V(\cdot ,t)$ are uniformly bounded, and the semiflow $\Phi (t)$ is point dissipative.

## Appendix C: Proof of Lemma 3.2

With the conclusion in Lemma 3.1, we denote by *ξ* the eigenfunction of the following eigenvalue problem:

C.1 $$ \textstyle\begin{cases} D_{2}\Delta \xi + \frac{ [(1-r(\cdot ))\beta (\cdot )S^{0}+(1-\eta (\cdot ))\frac{\alpha (\cdot )V^{0}}{K(\cdot )} ] \xi}{\Re _{0}}-( \gamma (\cdot )+d_{2}(\cdot ))\xi =0, \\ \frac{\partial I}{\partial \nu}=0. \end{cases} $$

Here we list the first equation of (C.1) separately:

C.2 $$ D_{2}\Delta \xi +\frac{1}{\Re _{0}} \biggl[ \bigl(1-r(\cdot )\bigr)\beta (\cdot )S^{0}+\bigl(1- \eta (\cdot ) \bigr)\frac{\alpha (\cdot )V^{0}}{K(\cdot )} \biggr]\xi -\bigl( \gamma (\cdot )+d_{2}( \cdot )\bigr)\xi =0. $$

Set $\delta ^{*}$ as the positive eigenfunction corresponding to $\lambda _{0}$, thus

C.3 $$ D_{2}\Delta \delta ^{*}+ \biggl[ \bigl(1-r(\cdot )\bigr)\beta S^{0}+\bigl(1-\eta (\cdot )\bigr) \frac{\alpha (\cdot )V^{0}}{K(\cdot )} \biggr]\delta ^{*}-\bigl(\gamma ( \cdot )+d_{2}(\cdot )\bigr)\delta ^{*}=\lambda _{0}\delta ^{*}. $$

Multiplying (C.2) by $\delta ^{*}$ and (C.3) by *ξ*, integrating them on Ω, then subtracting, we have

$$ \biggl(1-\frac{1}{\Re _{0}}\biggr) \int _{\Omega}\biggl[\bigl(1-r(\cdot )\bigr)\beta (\cdot )S^{0}+ \frac{(1-\eta (\cdot ))\alpha (\cdot )V^{0}}{K(\cdot )}\biggr]\xi \delta ^{*}\,dx = \lambda _{0} \int _{\Omega}\xi \delta ^{*}\,dx . $$

Since both sides of integrals are positive, thus $1-\frac{1}{\Re _{0}}$ and $\lambda _{0}$ have the same sign.

## Appendix D: Proof of Lemma 3.3

With the equation of *I* in (2.2), with the maximum principle and Hopf boundary lemma, for $t^{*}$ and $I(\cdot ,t^{*},\phi )\not \equiv 0$, we have $I\geq \tilde{I}$, where *Ĩ* satisfies

D.1 $$ \textstyle\begin{cases} \frac{\partial \tilde{I}}{\partial t} =D_{2}\Delta I-(\gamma (\cdot )+d_{2}( \cdot ))I,\quad (x,t)\in \Omega \times [0,\infty ), \\ \frac{\partial \tilde{I}}{\partial \nu} =0,\quad x\in \partial \Omega . \end{cases} $$

With Lemma 2.2, which implies $I(\cdot ,t,\phi )>0$. Thus from the equation of *S* and *V* in (2.2) we have *S*, *V* satisfies

D.2 $$ \textstyle\begin{cases} \frac{\partial S}{\partial t}\geq D_{1}\Delta S+\Lambda _{*}S-(r^{*}+(1-r_{*}) \beta ^{*}I^{*}+d^{*}_{1})S,\quad (x,t)\in \Omega \times [0,\infty ), \\ \frac{\partial V}{\partial t}\geq D_{3}\Delta V+r_{*}S-((1-\eta _{*}) \alpha ^{*}I^{*}+\eta ^{*}+d_{3}^{*})V,\quad (x,t)\in \Omega \times [0, \infty ), \\ \frac{\partial \tilde{S}}{\partial \nu}= \frac{\partial \tilde{V}}{\partial \nu}=0,\quad x\in \partial \Omega . \end{cases} $$

By applying the comparison principle, we can infer that

$$ \liminf_{t\to \infty}S(\cdot ,t,\phi )\geq \frac{\Lambda _{*}}{r^{*}+(1-r_{*})\beta ^{*}I^{*}+d^{*}_{1}} $$

and

$$ \liminf_{t\to \infty}V(\cdot ,t,\phi )\geq \frac{r_{*}\Lambda _{*}}{[r^{*}+(1-r_{*})\beta ^{*}I^{*}+d^{*}_{1}][(1-\eta _{*})\alpha ^{*}I^{*}+(\eta ^{*}+d^{*}_{3})]}, $$

which completes the proof.
